# Effect of ZrC Nanopowders on Enhancing the Hydro/Dehydrogenation Kinetics of MgH_2_ Powders

**DOI:** 10.3390/molecules26164962

**Published:** 2021-08-17

**Authors:** Mohamed Sherif El-Eskandarany, Naser Ali, Fahad Al-Ajmi, Mohammad Banyan

**Affiliations:** Nanotechnology and Applications Program, Energy and Building Research Center, Kuwait Institute for Scientific Research, Safat 13109, Kuwait; nmali@kisr.edu.kw (N.A.); ftajmi@kisr.edu.kw (F.A.-A.); mbanyan@kisr.edu.kw (M.B.)

**Keywords:** hydrogen energy, hydrogen storage, light metal hydrides, reactive ball milling, refractory metal, thermal stability, hydrogenation/dehydrogenation kinetics, cycle lifetime

## Abstract

Hydrogen has been receiving great attention as an energy carrier for potential green energy applications. Hydrogen storage is one of the most crucial factors controlling the hydrogen economy and its future applications. Amongst the several options of hydrogen storage, light metal hydrides, particularly nanocrystalline magnesium hydride (MgH_2_), possess attractive properties, making them desired hydrogen storage materials. The present study aimed to improve the hydrogen storage properties of MgH_2_ upon doping with different concentrations of zirconium carbide (ZrC) nanopowders. Both MgH_2_ and ZrC were prepared using reactive ball milling and high-energy ball milling techniques, respectively. The as-prepared MgH_2_ powder was doped with ZrC (2, 5, and 7 wt%) and then high-energy-ball-milled for 25 h. During the ball milling process, ZrC powders acted as micro-milling media to reduce the MgH_2_ particle size to a minimal value that could not be obtained without ZrC. The as-milled nanocomposite MgH_2_/ZrC powders consisted of fine particles (~0.25 μm) with a nanosized grain structure of less than 7 nm. Besides, the ZrC agent led to the lowering of the decomposition temperature of MgH_2_ to 287 °C and the reduction in its apparent activation energy of desorption to 69 kJ/mol. Moreover, the hydrogenation/dehydrogenation kinetics of the nanocomposite MgH_2_/ZrC system revealed a significant improvement, as indicated by the low temperature and short time required to achieve successful uptake and release processes. This system possessed a high capability to tackle a long continuous cycle lifetime (1400 h) at low temperatures (225 °C) without showing serious degradation in its storage capacity.

## 1. Introduction

The global use of fossil fuels has increased drastically as people’s standard of living has improved. Climate change, driven by increasing carbon dioxide emissions, will wreak havoc on society [1]. The threat of pollution will be exacerbated by the forced expansion of using low-grade fossil fuels. Improved living standards will raise the demand for chemicals derived from fossil fuels, which will be used to make higher-value end-use commodities such as plastics and pharmaceuticals. Due to the severe rivalry over dwindling supplies, the price of energy and chemicals derived from fossil fuels will increase. Due to environmental and health points of view, fossil fuels emit carbon dioxide and other harmful air pollutants such as SO_2_ and NO_x_ when burned [2].

The depletion of fossil fuels is a major motivator for finding clean alternative sources to power energy systems. As a result, using renewable and sustainable energy sources such as solar, wind, and geothermal energy to produce clean and efficient energy systems has become one of the world’s hottest research subjects, drawing a large number of academics and researchers [3].

Among the different green-energy options, hydrogen, which possesses a high energy density, has been considered the most suitable carbon-free energy carrier that can be used as an alternative to fossil fuel [4]. The convenience, safety, and versatility of hydrogen as an energy carrier are linked to its unique features of being easy to manufacture from a renewable energy system and convert to a desired form of energy [5]. One of the key advantages of hydrogen is that when burned, CO_2_ is not produced. Fuel cells (FCs) that run on hydrogen offer a wide range of potential uses, ranging from a few watts to gigawatts. Furthermore, compared to an internal combustion engine, hydrogen has the ability to drive a fuel-cell engine more efficiently [6]. The FCs could supply auxiliary power for electrical appliances such as air conditioning and refrigerators in mobile applications [7]. For automobile [8] and vehicle powertrain applications [9], hydrogen-polymer electrolyte membrane (PEM) FCs have become a well-known option [10].

The hydrogen economy and its potential applications are influenced by three variables. Apart from hydrogen generation and transportation, hydrogen storage is seen to be the most important aspect influencing the possible usage of hydrogen in real-world applications. Metal hydrides, notably MgH_2_, have very appealing features as compared to typical hydrogen storage systems. This system has a higher hydrogen storage density of 6.5 H atoms/cm^3^ for MgH_2_ when compared with pure hydrogen gas (0.99 H atoms/cm^3^) or liquid hydrogen (4.2 H atoms/cm^3^). Mg metal has various advantages for hydrogen storage, including low cost, light weight, and high gravimetric (7.60 wt%) and volumetric (110 g/L) hydrogen storage capacities [11,12,13,14,15,16,17,18]. In spite of the attractive and useful properties of Mg/MgH_2_, there are some serious drawbacks that should be solved before using this system for FC-hydrogen storage applications. The tetragonal-MgH_2_ (β-phase) is thermodynamically very stable (H^for^ = −75 kJ/mol. H_2_) and decomposes at a high temperature of 350 °C [19]. Furthermore, it has a high apparent activation energy (above 130 kJ/mol) and very slow hydrogenation/dehydrogenation kinetics under 325 °C [20].

Within the last three decades, great efforts have been dedicated in order to improve the hydrogen storage behavior of MgH_2_, using mechanical treatment and catalyzation approaches. Long-term high-energy ball milling [21], cold-rolling [22,23], equal channel angular pressing [24], and high-pressure torsion [25,26] are some techniques used to introduce severe plastic deformation to the Mg lattice, leading to the destabilization of the β-MgH_2_ phase that tends to transform into a less stable phase (γ-MgH_2_) with desired kinetics of hydrogen uptake/release.

Besides the mechanically enhanced approach, a different scenario has been used to improve the behavior of MgH_2_, using a long list of catalytic agents: pure transition metals such as Ni, Ti, V, and Nb (see, for example, [27,28,29,30]) and their alloys (e.g., TiV [31], CrTi [32], TiMn_2_ [33], VTiCr [34], and ZrNi_5_ [17]). Doping MgH_2_ with different concentrations of such reactive homogeneous and heterogeneous metallic catalysts led to significant improvements in the hydrogenation/dehydrogenation behavior of MgH_2_. Besides, a new category of metastable metallic alloys, such as big-cube Zr_2_ Ni [35] and Zr_70_ Ni_20_ Pd_10_ metallic glass [18] that were successfully used as catalytic agents, led to outstanding improvements in the hydrogen storage properties of MgH_2_. Additionally, different families of refractory metal compounds, including oxides (Nb_2_O_5_ [8], Cr_2_O_3_ [36], TiO_2_ [37], and La_2_O_3_ [38]), hydrides (TiH_2_ [39], LaH_3_ [40], and NbH [40]), as well as carbides (e.g., SiC [41] and TiC [42]), have been efficiently employed to improve the poor kinetics of MgH_2_ powders. Within the last decade, some advanced nanocarbon materials, such as single-walled carbon nanotubes [43] and graphene nanofibers [44], have shown remarkable beneficial effects on changing the hydrogen storage properties of Mg/MgH_2_. In 2021, a novel catalyzation process was proposed to enhance the thermodynamics and kinetics behaviors of Mg metal using Ni powders via a cold spray process technique [45].

In the present study, we attempted to improve the kinetics of the uptake/release of MgH_2_ powders, using a new catalytic agent of ZrC. This compound was selected according to its great hardness value. It can then be expected that upon ball milling with Mg/MgH_2_ powders, ZrC can play a great role as micro-milling media for reducing the particle size of the base material, leading to the enhancement of the kinetic characteristics of the hydride phase. Besides, ZrC with its high thermal stability would not decompose during the hydrogenation process of Mg metal. Moreover, ZrC does not react with Mg and/or MgH_2_ to form any undesired reactive or intermediate compounds. Thus, it can be safely used as a heterogenous catalytic agent without the drastic decrease in the hydrogen storage capacity of Mg. The present work was undertaken in part to introduce a new nanocomposite system of MgH_2_-based materials that has an excellent performance of cycle lifetime with fast hydrogenation/dehydrogenation kinetics.

## 2. Results and Discussions

### 2.1. Structural Analysis and Morphology

#### 2.1.1. MgH_2_ Nanocrystalline Powders

**Figure 1:**
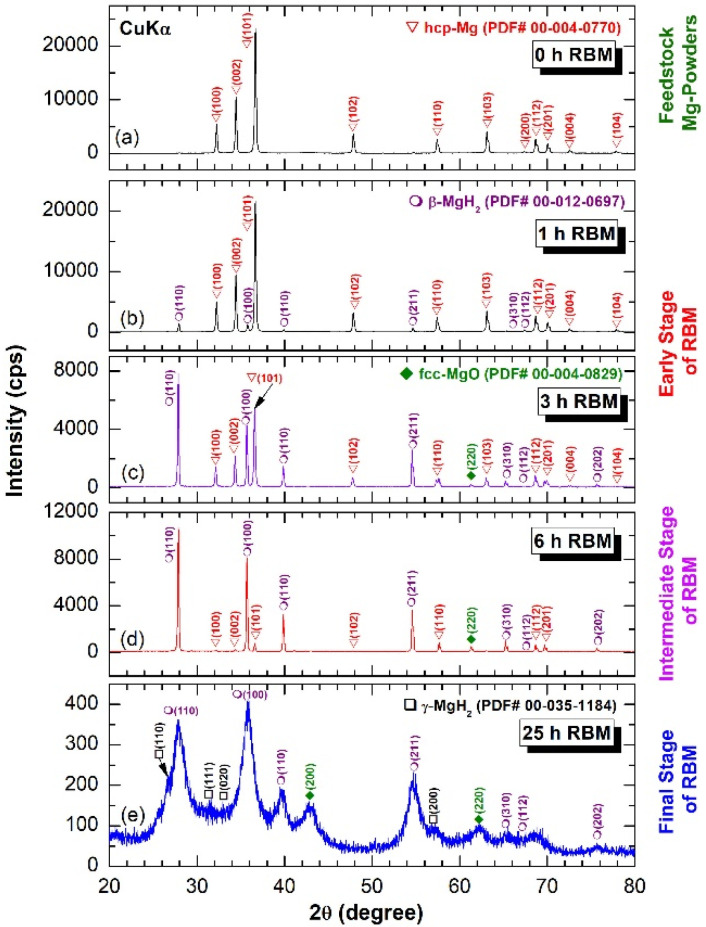
*X*-ray diffraction (XRD) pattern of as received hcp Mg powder is displayed in (**a**), where the XRD patterns of the powders obtained after reactive ball milling (RBM) for 1, 3, 6 and 25 h are presented in (**b**–**e**), respectively.

**Figure 2:**
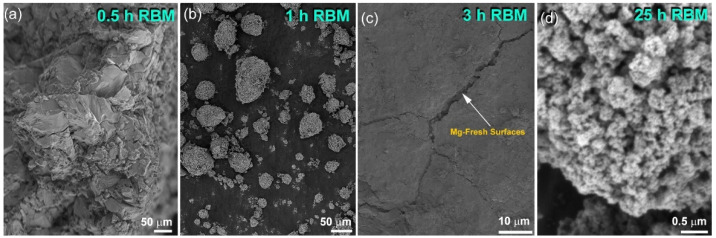
Field-emission scanning electron microscope (FE-SEM) micrographs of Mg powders obtained after RBM under pressurized hydrogen for (**a**) 0.5, (**b**) 1, (**c**) 3 and (**d**) 25 h.

An *X*-ray technique was employed to monitor the progress of the gas–solid reaction undertaken between Mg powders and H_2_, using the reactive ball milling (RBM) method. The general crystal structural changes in hcp Mg and the formation of new phases were investigated after the early (1–3 h), intermediate (3–6 h), and final stages of the RBM time. The *X*-ray diffraction (XRD) pattern of the starting feedstock powder, which is displayed in Figure 1a, displayed sharp Bragg-diffraction peaks related to hcp Mg (PDF # 00-004-0770). After a few minutes (30 min) of RBM, the powders were agglomerated due to the effect of cold welding created by the ball–powder–ball collisions, as displayed in Figure 2a. These agglomerated powders tended to disintegrate into small aggregates (~20–180 μm in diameter) upon the increase in the RBM time to 1 h, as shown in Figure 2b. The corresponding diffractogram of the disintegrated powders at this stage of RBM time is shown in Figure 1b.

Besides the Bragg peaks of unreacted hcp peaks, low-intensity diffracted lines were detected, suggesting the formation of the reacted β-MgH_2_ phase (PDF # 00-012-0697), as presented in Figure 1b. At the intermediate stage of RBM (3–6 h), the large Mg aggregates were disintegrated along their grain boundaries to form smaller grains with new active surfaces, as displayed in Figure 2c. Initiation of these new surfaces promoted a forward gas–solid reaction and the formation of a reactive MgH_2_ phase. This is implied by the formation of pronounced high-intensity Bragg lines, which corresponded to the β-MgH_2_ phase (Figure 1c). The formation of larger volume fractions of fresh surface Mg powders upon increasing the RBM time (6 h) enhanced the reaction between the diffusion couples (Mg and H_2_), leading to increases in the volume fraction of the reacted phase against pure Mg metal. Accordingly, the Bragg peaks related to hcp Mg were hardly detected in the diffractogram related to the powders obtained after 6 h of RBM (Figure 1d).

**Figure 3:**
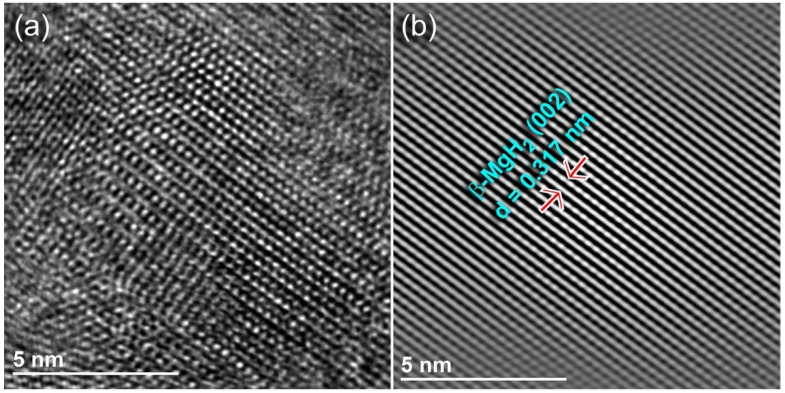
(**a**) Field emission high-resolution transmission electron microscope (FE-HRTEM) image of MgH_2_ powders obtained after 18 h of RBM. The filtered-atomic-scale TEM image of the fringe image in (**a**) is present in (**b**).

Severe lattice imperfections were continuously developed in the MgH_2_ lattice upon increasing the RBM time to 18 h, as indexed by the staking faults appearing in β-MgH_2_ (002), as shown in Figure 3a,b. The generation of these lattice imperfections led to further disintegration of the MgH_2_ powders and the formation of nanocluster powder particles after 25 h (Figure 2d). The XRD pattern of this end-product revealed significant broadening, obtained as a result of the grain refinement and lattice strain, as displayed in Figure 1e. The existence of metastable γ-MgH_2_ (PDF# 00-035-1184) was produced due to distortion of the most stable phase of β-MgH_2_ (Figure 1e).

#### 2.1.2. ZrC Catalytic Agent Nanopowders

**Figure 4:**
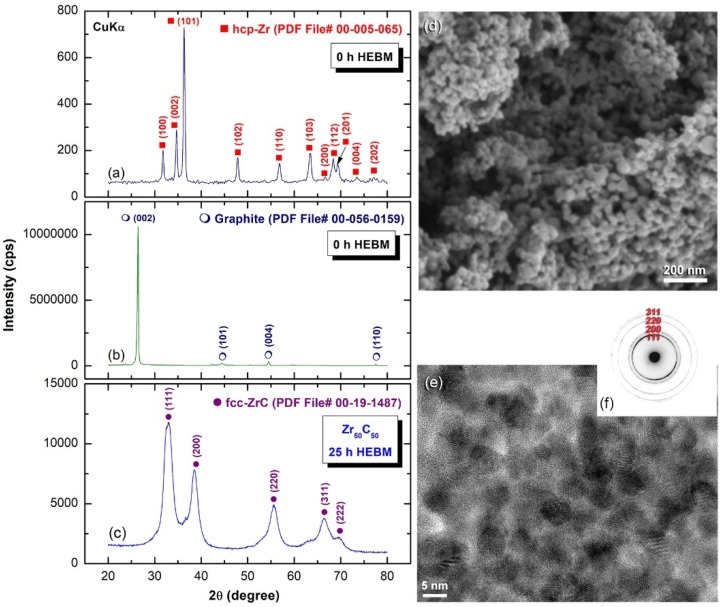
XRD patterns of (**a**) as-received hcp-Zr and (**b**) graphite powders. The XRD pattern of equiatomic fcc-ZrC nanopowders obtained after 25 h of high-energy ball milling (HEBM) is displayed in (**c**). The morphological characteristics of as-prepared fcc-ZrC powders are indexed in (**d**) and (**e**) by FE-SEM and FE-HRTEM, respectively. The nanobeam diffraction pattern (NBDP) taken from the middle zone of (**e**) is presented in (**f**).

For the present work, equiatomic nanocrystalline fcc-ZrC powders were synthesized from elemental Zr and graphite powders, using a high-energy ball mill (HEBM). Figure 4a,b display the XRD patterns for the starting feedstock powders of hcp Zr (PDF File# 00-005-065) and hcp-C (PDF File# 00-056-0159), respectively. A new phase was detected after 25 h of HEBM, as indexed in the XRD pattern shown in Figure 4c. Analysis of the diffracted lines that matched well with PDF File# 00-19-1487 indicated the formation of a single phase of fcc-ZrC. The HEBM ZrC powders had a cluster-like morphology composed of spherical nanoparticles with a diameter of less than 50 nm (Figure 4d). The field emission high-resolution transmission electron microscope (FE-HRTEM) image indicated the formation of uniform nanospheres in the range between 2 and 7 nm in diameter, as shown in Figure 4e. Moreover, the corresponding nanobeam diffraction pattern (NBDP), implied the formation of fcc ZrC, characterized by (111), (200), (220) and (311), as displayed in Figure 4f. The absence of sharp spots from the Debye–Scherrer rings implied the existence of nanocrystalline ZrC powders.

#### 2.1.3. Nanocomposite MgH_2_/x-ZrC (x; 2, 5 and 7 wt.%) Powders

**Figure 5:**
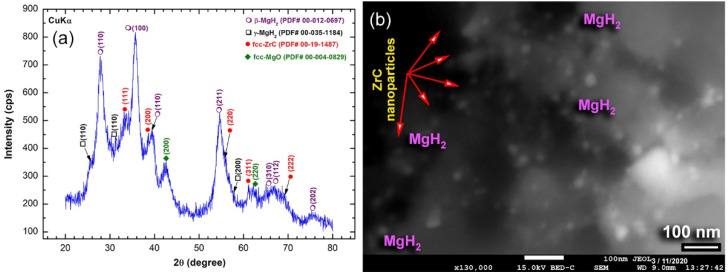
(**a**) XRD pattern and (**b**) back-scattering electron FE-SEM micrograph of mechanically mixed MgH_2_/5 wt.% ZrC nanocomposite powders obtained after 50 h of HEBM. The nanodispersoid ZrC, which are indexed by the red arrow labels in (**b**), were embedded into the MgH_2_ powder matrix.

The ZrC nanoparticles obtained after 25 h of milling were used to improve the hydrogen storage characteristics of MgH_2_ powders upon mechanically induced mixing for 50 h with different mass fractions (2, 5, and 7 wt.%), using HEBM. The XRD pattern of nanocomposite MgH_2_/5 wt.% ZrC powders obtained after 50 h of HEBM is displayed in Figure 5a. The diffractogram possessed broad Bragg peaks of the base MgH_2_ powders and the modifier agent of ZrC, as presented in Figure 5a. Both phases kept their existence after milling for 50 h with no evidence for the formation of any reacted phase.

**Figure 6:**
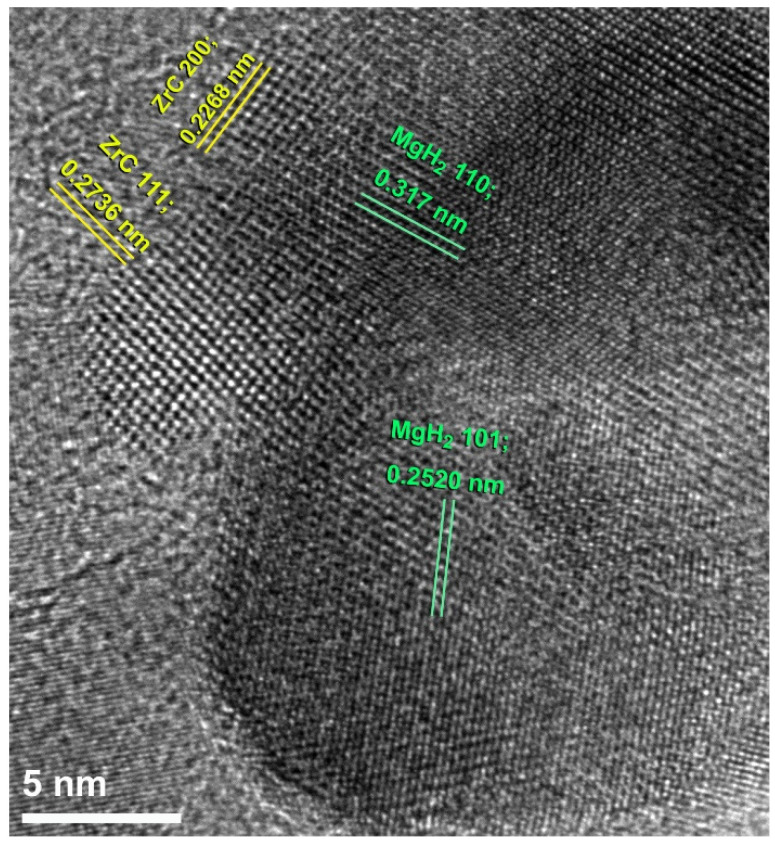
FE-HRTEM with atomic resolution of nanocomposite MgH_2_/5 wt.% ZrC powders obtained after 50 h of HEBM.

The atomic-resolution TEM image of the nanocomposite powders obtained after 50 h of milling is presented in Figure 6. The figure shows two individual MgH_2_ particles, indexed by β-MgH_2_ (110) and (101). Meanwhile, two ZrC nanoparticles, characterized by fcc -ZrC (111) and (200), were adhered to the MgH_2_ particles, as displayed in Figure 6. Examining the local structure of the interfaces between these two different materials beyond the atomic level suggested the absence of any reacted phase(s). This implies that ZrC was natural during milling with MgH_2_ powders and played the role of a heterogeneous catalyst. It is worth mentioning that the positions of the Bragg peak for MgH_2_ shifted to the low-angle side, suggesting the expansion of the lattice parameter without altering the crystal.

Besides, the Bragg lines of MgH_2_ had become broader, when compared with the original diffractogram of as-prepared MgH_2_ powders (Figure 1e). This may suggest the synergetic effect of further milling with ZrC hard powders (Figure 5a). The back scattering electron micrograph, using a 15 kV FE-SEM with a BSE detector (BED) for the nanocomposite MgH_2_/5 wt.% ZrC, obtained after 25 h of HEBM, is shown in Figure 5b. The powders were composed of MgH_2_ matrix, where spherical ZrC nanoparticles (<10 nm) were embedded uniformly into the matrix.

**Figure 7:**
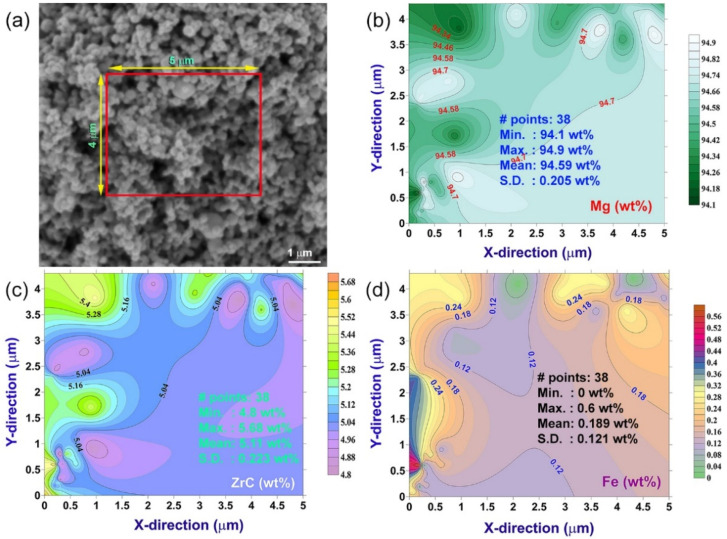
(**a**) FE-SEM micrograph of mechanically mixed MgH_2_/5 wt% ZrC nanocomposite powders obtained after 50 h of HEBM. The indexed rectangular zone (4 cm × 5 cm) shown in (**a**) refers to the analytical area, in which intensive EDS analysis was conducted. The corresponding isochemical contour maps of Mg, ZrC, and Fe contamination are displayed in (**b**–**d**), respectively.

The distribution of ZrC nanoparticles in MgH_2_ powders after processing for 25 h of HEBM was investigated by intensive EDS analysis, using FE-SEM. Figure 7a displays a FE-SEM micrograph of nanocomposite MgH_2_/5 wt.% ZrC powder obtained after 25 h of milling. The indexed rectangular zone (5 μm × 4 μm), shown in the middle part of the micrograph, refers to the selected area chosen for EDS analysis. The area was classified into subrectangular zones (0.5 μm × 0.4 μm), where the analysis was conducted in the middle of each subzone (Figure 7a). In general, the powder obtained after this stage of HEBM possessed a narrow distribution particle size range (100–300 nm) with a spherical-like morphology, as shown in Figure 7a. We should emphasize that refractory ZrC nanopowders played the role of micro-milling media and led to drastic grain refining of the MgH_2_ powders.

In addition, further ball milling time (50 h) promoted a homogeneous distribution of nanograined ZrC powders into the metal hydride matrix. The compositional analysis obtained from EDS measurements was employed to design isochemical contour maps for Mg (Figure 7b), ZrC (Figure 7c), and Fe (Figure 7d). The Fe was introduced to the nanocomposite powders due to using tool-steel milling tools. The contour intervals were selected to cover all concentrations of Mg (94.34–94.7 wt.%), ZrC (5.04–5.4 wt.%), and Fe (0.12–0.24 wt.%), as shown in Figure 7b–d, respectively.

No obvious degradation in ZrC concentration or drastic compositional gradient could be detected, suggesting a uniform distribution of ZrC nanoparticles into the MgH_2_ matrix. The average concentrations of Mg and ZrC were 94.9 wt.% (Figure 7b) and 5.11 wt.% (Figure 7c), respectively. The powders, however, were contaminated with about 0.19 wt.% of Fe (Figure 7d).

### 2.2. Thermal Stability

**Figure 8:**
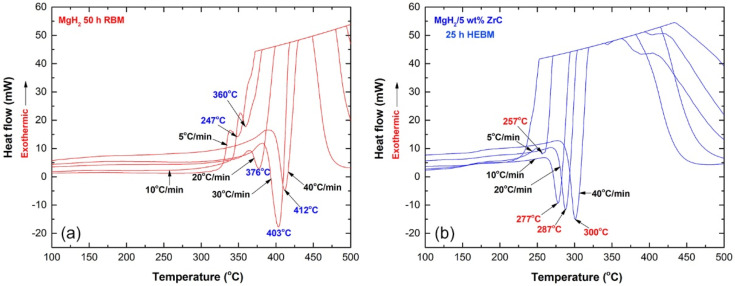
DSC thermograms conducted at heating rates of 5, 10, 20, 30, and 40 °C/min for (**a**) MgH_2_ powders, obtained after 50 h of RBM, and (**b**) mechanically mixed MgH_2_/5 wt% ZrC nanocomposite powders obtained after 25 h of HEBM.

**Figure 9:**
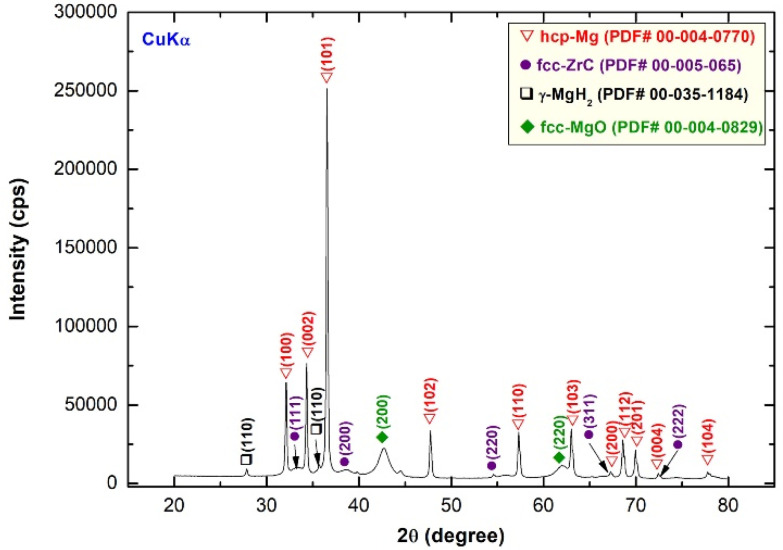
XRD pattern of nanocomposite MgH_2_/5 wt.% ZrC milled for 25 h of HEBM and then heated up to 500 °C in a DSC under the flow of He gas.

Atmospheric helium-pressure DSC was employed to investigate the thermal stability, indexed by decomposition temperature (T_p_) and activation energy (E_a_), for synthesized nanocrystalline MgH_2_ before and after doping with ZrC nanoparticles. The DSC thermogram of MgH_2_ nanopowders, obtained after 50 h of RBM, are displayed in Figure 8a with different heating rates (k) of 5, 10, 20, 30, and 40 °C/min. All the scans revealed single endothermic events related to the decomposition of the MgH_2_ phase. This is implied by the XRD pattern of the sample heated up to 500 °C, which revealed sharp Bragg peaks related to pure Mg and fcc ZrC overlapped with an undecomposed minor volume fraction of MgH_2_, as displayed in Figure 9. While the peak height increased proportionally with the increase in k, T_p_ significantly shifted to the higher-temperature side upon the increase in the heating rates from 5 to 40 °C/min, as shown in Figure 8a. The as-synthesized MgH_2_ nanopowders revealed a high decomposition temperature, exemplified by the T_p_ measured at 10 °C/min (360 °C), as shown in Figure 8a.

The improved dehydrogenation kinetics, measured under a helium gas atmosphere, was evaluated by calculating the E_a_ of the decomposition reaction for both pure MgH_2_ and corresponding MgH_2_/5 wt.% ZrC samples. In the present work, the activation energy for dehydrogenation was investigated according to the Arrhenius Equation (1):

E_a_ = −RT_p_ ln(k/k_0_) (1)

where k is a temperature-dependent reaction rate constant, R is the gas constant, and Tp is the absolute temperature. The value E_a_ of the reaction was determined by measuring the decomposition of T_p_ corresponding to the different heating rates (k) and then plotting ln(k) versus 1/T_p_, where the E_a_ of synthesized MgH_2_ nanopowders was 123 kJ/mol, and the E_a_ obtained for nanocomposite MgH_2_/5 wt.% ZrC was 69 kJ/mol. This indicates a significant destabilization of the MgH_2_ upon doping with 5 wt.% ZrC.

### 2.3. Pressure-Composition-Temperature

**Figure 10:**
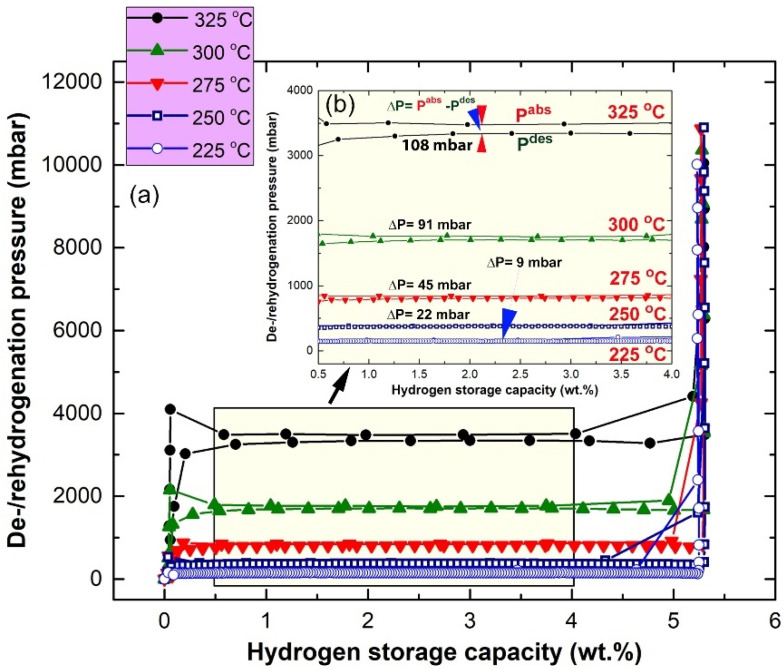
Pressure-composition-temperature (PCT) curves conducted at 225, 250, 275, 300 and 325 °C for nanocomposite MgH_2_/5 wt.% ZrC obtained 25 h of HEBM. (**a**) Sievert’s approach at different temperatures (**b**) wide pressure gaps (∆P) between the hydrogenation (P^abs^) and dehydrogenation (P^des^) plateaus.

Pressure–composition–temperature (PCT), also known as pressure–composition isotherms (PCI), is the most basic measurement, used to investigate the thermodynamic properties of a hydrogen storage system. The PCT correlations of nanocomposite MgH_2_/5 wt.% ZrC powders, obtained after 25 h of HEBM, were volumetrically investigated by Sievert’s approach at different temperatures of 225, 250, 275, 300 and 325 °C, as displayed in Figure 10a. The PCT measurements were started by introducing H_2_ to the system at the desired temperature, and then the system was closed while the sample and gas reacted under isochoric conditions. All PCT experiments were achieved, using the run-of-RBM (raw-nanocomposite powders), before conducting powder activation. This was necessary in order to realize the original shape of the PCT curve, and its slope. In all applied temperatures, single reversible hydrogenation/dehydrogenation cycles were characterized, with the absence of wide pressure gaps (∆P) between the hydrogenation (P^abs^) and dehydrogenation (P^des^) plateaus, as shown in Figure 10b. Accordingly, the near values of ∆P (∆P = P^abs^ − P^des^) indicate the absence of the hysteresis phenomenon for this hydrogen storage nanocomposite system, as can be realized in Figure 10a,b.

**Figure 11:**
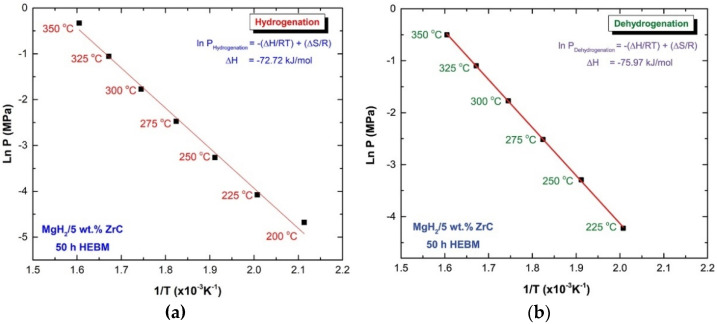
Van’t Hoff plot of the plateaus shown respectively in Figure 10 a for hydrogenation (**a**) and 10b dehydrogenation (**b**) of nanocomposite MgH_2_/5 wt.% ZrC obtained after 25 h of HEBM.

Moreover, the presence of single clear hydrogenation/dehydrogenation plateaus can be seen in the range between 0.5 and 4 wt.% H_2_ at all temperature ranges (Figure 10a). Smooth plateaus of hydrogen uptake/release were characterized in the whole hydrogen concentrations range (0.25–5.25 wt.% H_2_) for all applied temperatures, as presented in Figure 10b. The hydrogen equilibrium pressure measurements were used in the present study to investigate the heat of hydrogen absorption, using the van’t Hoff Equation (2):

(2) $$\ln(\frac{P_{eq}}{P_{o}})=-\left(\frac{∆H}{RT}+\frac{∆S}{R}\right)$$

where *P_eq_* is the hydrogen pressure under equilibrium at a given specific temperature, *T*; *P_o_* is a reference pressure of 1 bar; *R* is the gas constant (0.0083145 J/K.mol); ∆*H* is the molar enthalpy of metal hydride formation (MgH_2_); and ∆*S* is the entropy of absorption. The ∆*H* of hydrogenation can be directly calculated from plotting the natural log of each *P_eq_* point versus the corresponding 1/*T*, as shown in Figure 11a.

In the present work, the calculated ∆*H* and ∆*S* for MgH_2_ doped with 5 wt.% ZrC were −72.74 kJ/mol and 112.79 J/mol H_2_/K, respectively. On the other hand, the strength of Mg–H bonds, which is indicated by the enthalpy of decomposition, can be calculated via the van’t Hoff approach, using the equilibrium dehydrogenation pressure in the PCT measurements. The ∆*H* of decomposition and the corresponding ∆*S* of the nanocomposite MgH_2_/5 wt.% ZrC were calculated for the slope of the line displayed in Figure 11b, and they were found to be 75.97 kJ/mol and 119.15 J/mol H_2_/K, respectively.

### 2.4. Hydrogenation/Dehydrogenation Kinetics

#### 2.4.1. MgH_2_ Nanocrystalline Powders

**Figure 12:**
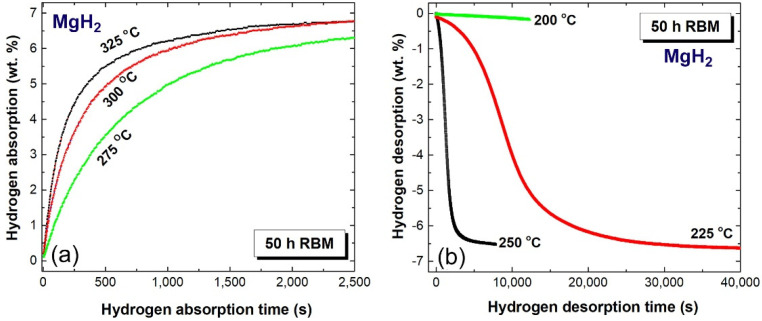
Kinetics measured at 275, 300 and 350 °C of (**a**) hydrogenation and (**b**) dehydrogenation of MgH_2_ powders obtained after 50 h of RBM.

Figure 12a,b display the synergetic effect of RBM time and applied temperature on the hydrogenation and dehydrogenation kinetics of RBM MgH_2_ powders obtained after 50 h of milling, respectively. As expected, the synthesized pure MgH_2_ powders exhibited poor hydrogenation/dehydrogenation kinetics, particularly at applied temperatures of less than 325 °C, as shown in Figure 12. For example, the times required to uptake and discharge ~6.5 wt.% H_2_ at 325 °C were 2500 s and 7800 s, as presented in Figure 12a,b, respectively. The absorption and desorption kinetics dropped significantly upon decreasing the temperature to 275 °C. This is indicated by the time necessary to absorb (~2500 s) and desorb (~40,000 s) 6.28 wt.% H_2_, as presented in Figure 12a,b, respectively.

#### 2.4.2. Nanocomposite MgH_2_/x-ZrC (x; 2, 5, and 7 wt.%) Powders

**Figure 13:**
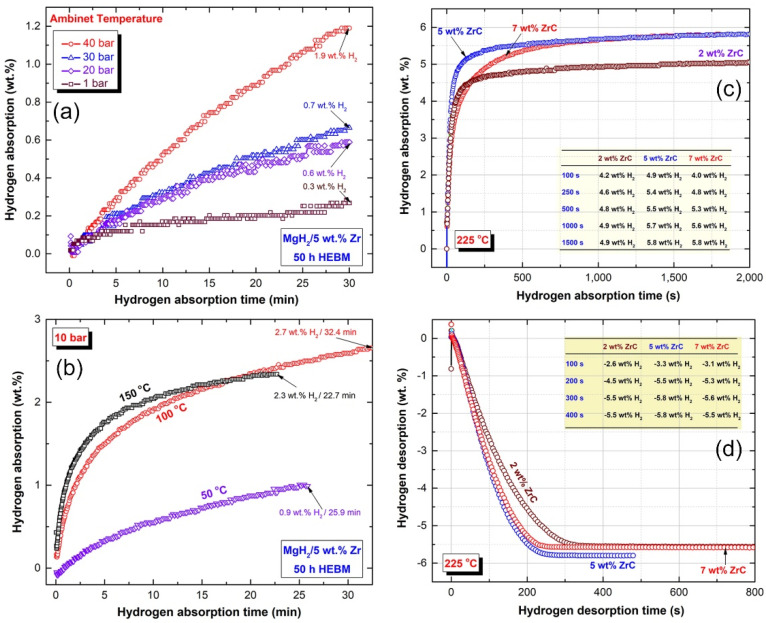
Hydrogenation kinetics of MgH_2_/5 wt.% ZrC system, measured at (**a**) ambient temperature under hydrogen pressures of 1, 20, 30, and 40 bar, and (**b**) 50, 100, and 150 °C under a hydrogen pressure of 10 bar. The hydrogenation and dehydrogenation kinetics measured at 225 °C under 10 and 0.04 H_2_ bar of MgH_2_/x-wt.% ZrC (x; 2, 5, 7) are presented in (**c**,**d**), respectively.

Figure 13 summarizes the hydrogenation/dehydrogenation kinetics obtained at selected temperatures upon mechanical mixing of nanocrystalline MgH_2_ powders with different concentrations (2, 5, and 7 wt.%) of ZrC nanopowder particles. The MgH_2_/5 wt.% ZrC system possessed excellent hydrogenation kinetics, suggested by its capability of absorbing 1.9, 0.7, 0.6, and 0.3 wt.% H_2_ at ambient temperature under pressures of 40, 30, 20, and 1 bar (Figure 13a).

Under an applied pressure of 10 bar, this system showed a good ability of absorbing 2.7 and 2.3 wt.% H_2_ within 32.4 and 22.7 min at low temperatures of 100 and 150 °C, respectively, as displayed in Figure 13b. It is worth mentioning that the system was able to absorb 0.9 wt.% H_2_ within 25.9 min at 50 °C under 10 bar, as shown in Figure 13b. Figure 13d displays the effect of ZrC nanopowder on improving the dehydrogenation kinetics of MgH_2_ powder. As shown in the figure, MgH_2_ doped with 2, 5 and 7 wt.% ZrC possessed excellent releasing kinetics, indicated by the relatively short time (200 s) needed to desorb about −4.5, −5.5, and 5.3 wt.%, respectively. After 400 s, the three systems approached saturated values in the range between −5.5 and −5.8 wt.% H_2_ (Figure 13d).

### 2.5. Cycle Lifetime

**Figure 14:**
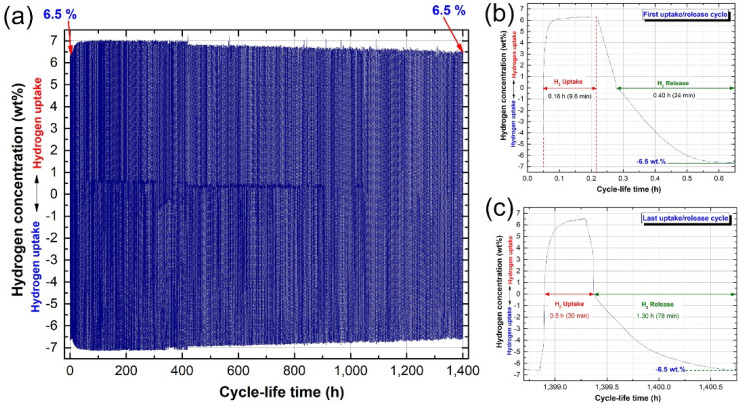
(**a**) Hydrogenation/dehydrogenation cycle lifetime of MgH_2_/5 wt% ZrC binary system, examined for 1400 h at 225 °C under uptake and hydrogen pressures of 10 and 0.4 bar, respectively. The hydrogenation/dehydrogenation cycles after the first and last cycles are presented in (**b**,**c**), respectively.

To obtain more information of the capability of the nanocomposite MgH_2_/5 wt.% ZrC system to perform a large number of uptake/release cycles without degradation, the powders were first activated at a higher temperature of 275 °C under hydrogenation/dehydrogenation hydrogen pressures of 35 and 0.2 bar, respectively. This activation step was required to improve the hydrogen storage capacity, which reached 6.9 wt.%, as shown in Figure 14a. During the thermal treatment (surface cleaning) under such high pressure and temperature, the bonding between Mg and O_2_ tended to break down. Accordingly, the yielded metallic Mg reacted with hydrogen to form MgH_2_, where the dissociated oxygen atoms combined with hydrogen to form a vapor of water molecules that were continuously evacuated outside of the system. As a result, the volume fraction of MgH_2_ was increased against MgO, leading to the enhancement of the hydrogenation/dehydrogenation kinetics (Figure 14a). More importantly, surface cleaning of MgH_2_ powders enhanced the kinetics of hydrogenation/dehydrogenation processes, as implied by the powder capability of achieving 1400 h (~60 days) of charging/discharging processes with the absence of failure, as displayed in Figure 14a. However, this long testing time led to obvious degradation in both the hydrogenation/dehydrogenation kinetics of the last cycle (Figure 14c) when compared with the first cycle (Figure 14b).

However, a marginal decrease (~less than 0.5 wt.% H_2_) in the storage capacity was detected after a compilation of 1400 h, as displayed in Figure 14a. This may be attributed to a moderate grain growth that occurred in MgH_2_ powders due to the long processing cycle lifetime. It was necessary to examine the morphological characteristics and local structure of powders after such a long cycle lifetime.

**Figure 15:**
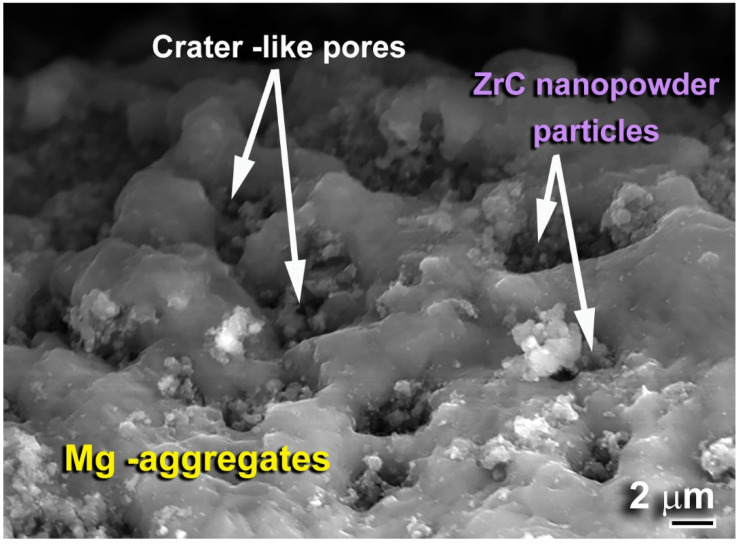
FE-SEM micrograph of MgH_2_/5 wt.% ZrC binary system, obtained after achieving 1400 h of cycle lifetime at 225 °C under uptake and hydrogen pressures of 10 and 0.4 bar, respectively (see Figure 14c).

The FE-SEM image of the cycled powders is presented in Figure 15. The powders were composed of dark-grey Mg aggregates with a rough surface, containing numerous numbers of pores with a crater-like morphology, as displayed in Figure 15. The presence of such pores facilitated successful hydrogen absorption/desorption. Besides, the ZrC nanopowders were embedded into the surface and subsurface of Mg metal through the pores to act as a grain growth inhibitor, as shown in Figure 15.

**Figure 16:**
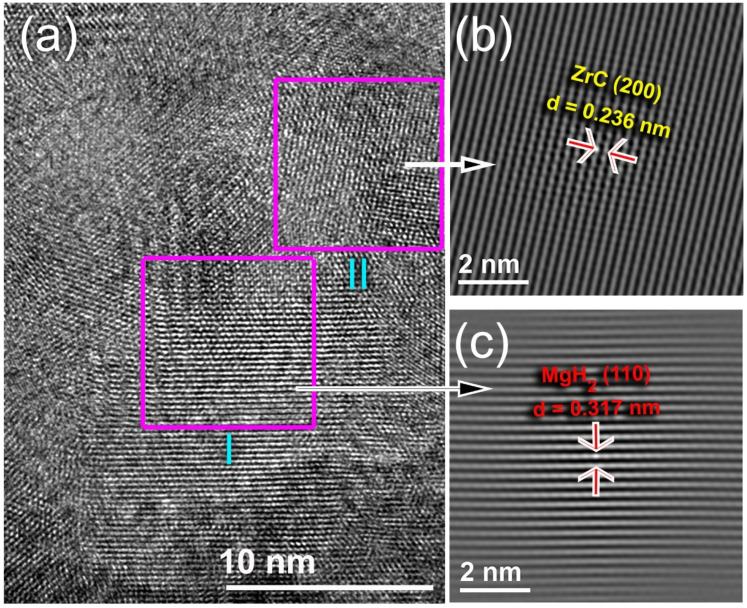
(**a**) FE-HRTEM image of MgH_2_/5 wt.% ZrC binary system, obtained after achieving 1400 h of cycle lifetime at 225 °C under uptake and hydrogen pressures of 10 and 0.4 bar, respectively. The corresponding filtered atomic resolution images taken for zones I and II are displayed in (**b**,**c**), respectively.

The local structure of the nanocomposite MgH_2_/5 wt% ZrC system obtained after a cycle lifetime for 1400 h was examined with atomic-resolution FE-TEM. The HRTEM image displayed fringe nanograins related to β-MgH_2_ (zone I in Figure 16a) oriented to the axis zone of (110), as displayed in Figure 16b. Besides, a ZrC nanograin (zone II in Figure 16a) was embedded into the MgH_2_ matrix, as indicated by the interplanar spacing (0.236 nm), which corresponds to ZrC (200), as presented in Figure 16c. It is worth mentioning that we could not detect any reacted phase such as pure hcp-Zr, fcc-ZrH_2_, and/or MgZr phases. This may suggest that ZrC nanopowder is a typical heterogeneous catalyst, where it does not react with MgH_2_ to form any intermediate phase.

Comparison of the present results indicates the capability of the MgH_2_/ZrC binary system to achieve a long cycle lifetime with excellent performance. For example, MgH_2_/Mn_2_Ti [33] and MgH_2_/10 wt% big-cube Zr_2_Ni [35] systems showed excellent performances for achieving about 1400 h; however, the process was undertaken at higher temperatures (250 °C to 275 °C) under a hydrogen pressure of 10 bar. These systems, however, had a lower hydrogen storage capacity of less than 5 wt%H_2_ and suffered from slower uptake/release kinetics. The advantages of using ZrC particles as a hydrogen storage modifier for the MgH_2_ binary system may be attributed to the abrasion effect of ZrC nanopowders, which led to a severe reduction in MgH_2_ powders upon HEBM. Moreover, the ZrC nanopowders played a critical role as a grain growth inhibitor that prevented MgH_2_ powders from severely increasing in size, thus overcoming any undesired kinetics degradation during the long cycle lifetime.

## 3. Materials and Methods

### 3.1. Materials Preparations

#### 3.1.1. Preparations of Nanocrystalline MgH_2_ Powders

Elemental powders of Mg (99.9 wt%, 80 μm) were provided by Alfa Aesar (CAS Number 7439-95-4), Kandel, Germany, and high-purity (99.999 wt.%) hydrogen gas was used as starting materials. A small amount (~5 g) of the powder was sealed into a tool steel vial (150 mL), using a GST (gas-temperature-monitoring system) inside a helium (He) gas atmosphere-glove box (UNILAB Pro Glove Box Workstation, mBRAUN, Daimlerstraße 29–31, D-76316 Malsch, Germany). A quantity of 50 tool steel balls (11 mm in diameter) were used as milling media. The ball-to-powder weight ratio was 42 to 1. The vial was then pressurized with 50 bar of hydrogen. The reactive ball milling was carried out at room temperature for 25 to 50 h with a rotation speed of 250 rpm, using a planetary-type ball mill (PM400) provided by RETSCH GmbH, Berlin, Germany.

#### 3.1.2. Preparations of ZrC Nanopowders

Elemental Zr (99.5 wt%, 20 μm, CAS Number7440-67-7) and graphite (99 wt.%, 20 μm, CAS Number7782-42-5) powders, provided by Alfa Aesar, Kandel, Germany, were employed as feedstock materials. The two powder species were balanced and mixed inside the glove box to obtain 5 g of equiatomic composition. The powders were then sealed in a tool steel vial (150 mL) together with 50 tool steel balls (11 mm in diameter), using a ball-to-powder weight ratio of 40 to 1. The system was mounted on the planetary-type ball mill (PM400), where the milling process started at room temperature for 25 h with a rotation speed of 250 rpm.

#### 3.1.3. Preparations of Nanocomposite MgH_2_/ZrC Nanopowders

The as-milled MgH_2_ powders were doped with the desired mass of as-prepared ZrC to obtain three nominal compositions of MgH_2_/x wt% ZrC (x; 2, 5 and 7) inside the glove box. The three composite systems were individually charged into tool steel vials (150 mL) and sealed together with 50 tool steel balls (11 mm), using 45:1 as the ball-to-powder weight ratio. The vial was then pressurized with 50 bar of hydrogen. The mechanically induced solid-state mixing was conducted at a milling speed of 250 rpm for 25 h, using a planetary-type ball mill (PM400).

### 3.2. Sample Characterizations

#### 3.2.1. Crystal Structure and Morphology

*X*-ray diffraction (XRD) with CuKα radiation was employed to investigate the average crystal structure of all samples, using the 9 kW Intelligent *X*-ray diffraction system, provided by SmartLab-Rigaku, Kawasaki, Japan. Then, a 200 kV-field-emission high-resolution transmission electron microscope (FE-HRTEM, JEOL-2100 F), supplied by JEOL, Chiba, Japan, was used to investigate the local structure of the synthesized powders. The morphological properties of the powders were studied by a 15 kV-field-emission scanning electron microscope (FE-SEM, JSM-7800 F/EDS, Chiba, Japan). The local composition of the as-prepared samples was investigated via FE-SEM/energy-dispersive *X*-ray spectroscopy (EDS, Oxford Instruments, Andor, 277.3 mi, Belfast, UK).

#### 3.2.2. Thermal Stability

The thermal stability of the samples was investigated by the Shimadzu Thermal Analysis System (TA-60 WS, Tokyo, Japan), using a differential scanning calorimeter (DSC) with different heating rates of 5, 10, 20, 30, and 40 °C/min. All DSC measurements were conducted under a flow (75 mi/min) of He.

#### 3.2.3. Hydrogenation/Dehydrogenation Kinetics

The absorption/desorption kinetics behavior of the samples were investigated via Sievert’s method, using PCTPro-2000, provided by Setaram Instrumentation, 12 Rue de Verdun, 69,300 Caluire-et-Cuire, France. An amount of 250–280 mg of the powders was handled and balanced to obtain the desired mass inside the He-glove box. The powders were then sealed into a vial made of chromium-resistance (Swagelok^®^) alloy inside the glove box. The vial was then inserted inside a portable standard autoclave Cu-clad holder. Prior to the kinetics measurements, the powders were activated at 350 °C under 35 bar of hydrogen overnight. Then, six volume calibrations were individually conducted at room temperature and at the desired measurement temperature.

## 4. Conclusions

Reactive ball milling was employed to fabricate nanocrystalline MgH_2_ powders in a gas–solid reaction fashion performed under high hydrogen pressure (~50 bar) for 25 h to 50 h. The hydrogenation/dehydrogenation kinetics of the as-prepared MgH_2_ powder was very slow and required the application of high temperature (above 325 °C) to absorb/desorb 6.5 wt.% H_2_ within 2500 s and 7800 s, respectively. Besides, the as-prepared MgH_2_ system obtained after 50 h of reactive ball milling possessed a high decomposition temperature of 376 °C at a heating rate of 20 °C/min with an apparent activation energy of 123 kJ/mol. To improve the hydrogen storage behavior of the as-prepared MgH_2_ binary system, ultrafine fcc-ZrC nanopowder was prepared via the carbonization reaction between metallic Zr and graphite powders, using a room-temperature high-energy ball mill. The as-prepared ZrC powders obtained after 25 h of milling possessed good morphological properties of being an ultrafine powder (less than 50 nm in diameter) with spherical-like nanograins, ranging between 2 and 7 nm in diameter. In the present work, MgH_2_ powders were doped with three individual concentrations of ZrC (2, 5 and 7 wt.%) and high-energy ball-milled for 25 h. The results showed that ZrC powders acted as micro-milling media to reduce the MgH_2_ particle size to a minimal value that could not be obtained without ZrC. After 25 h of milling, the hard ZrC nanopowders were embedded into the MgH_2_ matrix to form nanocomposite MgH_2_/ZrC powders that had an homogeneous composition and fair distribution of ZrC particles beyond the nano-level. The ZrC agent led to the minimization of the decomposition temperature (287 °C) of MgH_2_ and lowering in the apparent activation energy of desorption for MgH_2_ to 69 kJ/mol. The hydrogenation/dehydrogenation kinetics of the nanocomposite MgH_2_/ZrC system revealed significant improvement, as indicated by the low temperature and short time required to achieve successful uptake and release processes. This system possessed a high capability of tackling a long continuous cycle lifetime (1400 h) at low temperature (225 °C) without showing serious degradation in its storage capacity.

## Data Availability

Not available.

## Sample Availability

Samples of the compounds are not available from the authors.
